# Liposomes in drugs, nutraceuticals, and cosmetics

**DOI:** 10.7717/peerj.21198

**Published:** 2026-07-10

**Authors:** Eleaneth Baltodano Viales, Gustavo Alonso Carazo Berrocal, Jorge Andrés Pacheco Molina, Fredy Barrantes Pérez, German Leonardo Madrigal Redondo

**Affiliations:** Instituto de Investigaciones Farmacéuticas (INIFAR), Facultad de Farmacia, University of Costa Rica, Montes de Oca, San José, Costa Rica

**Keywords:** Lipidic bilayers, Phospholipid membranes, Liposomes, Cosmetics, Drugs, Nutraceuticals, Bioavailability

## Abstract

**Background:**

Liposomes are spherical vesicular structures formed by lipid bilayers with a hydrophilic internal nucleus. The size varies from about 20 nanometers to several micrometers. They were initially described in the sixties by the biophysicist Alec Bangham. Since then, multiple studies have been conducted to characterize them according to the number of layers: unilamellar, bilayers, multilamellar vesicles, and vesicles containing more vesicles called multivesicular vesosomes; size: between 0.025 µm and 2.5 µm, and functionality: Drug delivery system, cosmetic, nutraceutics. Moreover, there are several conventional and new methods for liposome preparation. For conventional liposomes, multiple technologies have been developed to enhance or impart specific characteristics, depending on the intended application of the liposomes, such as long-circulation liposomes, cationic liposomes, immunoliposomes, and specific stimulus-sensitive liposomes. In the medical field, liposomes are used in many routes of administration. Parenteral routes include most approved liposomal drugs; oral drugs have benefited from improved bioavailability. Pulmonary routes have emerged as an alternative to increase residence time and, therefore, therapeutic efficacy. Ocular routes are widely studied because liposomes are biodegradable and biocompatible. Nutraceuticals have emerged as an alternative or complementary therapy to treat many diseases, and liposomes enhance the bioavailability of these molecules. Liposomes also benefit the cosmetic industry by encapsulating substances, enhancing permeability, and enabling controlled release.

**Methodology:**

A bibliographic review was carried out by consulting databases such as ScienceDirect, PubMed, Wiley Online Library, Multidisciplinary Digital Publishing Institute (MDPI), National Center for Biotechnology Information (NCBI), Directory of Open Access Journals (DOAJ), and Nature. The keywords used were liposomes, liposomes and cosmetics, liposomes and cosmeceuticals, liposomes and nanotechnology, Transferosomes^®^, Ethosomes, liposomes-modified vesicles, liposomes and nutraceuticals, liposomes and natural products. The search was conducted in both languages, English and Spanish, covering the period from 2010 to 2025, and included all records available during that time.

**Results and Conclusion:**

These vesicles have a particular scientific relevance in the pharmaceutical, nutraceutical, and cosmetic fields. They are promising vehicles or carriers for substances and compounds that are unstable under specific conditions and can be easily degraded or have undesirable taste and smell. However, despite their advantages, challenges remain, including low solubility, limited half-life, oxidation, and hydrolysis of the phospholipids that form their membranes, and elevated production costs.

## Introduction

**Figure 1:**
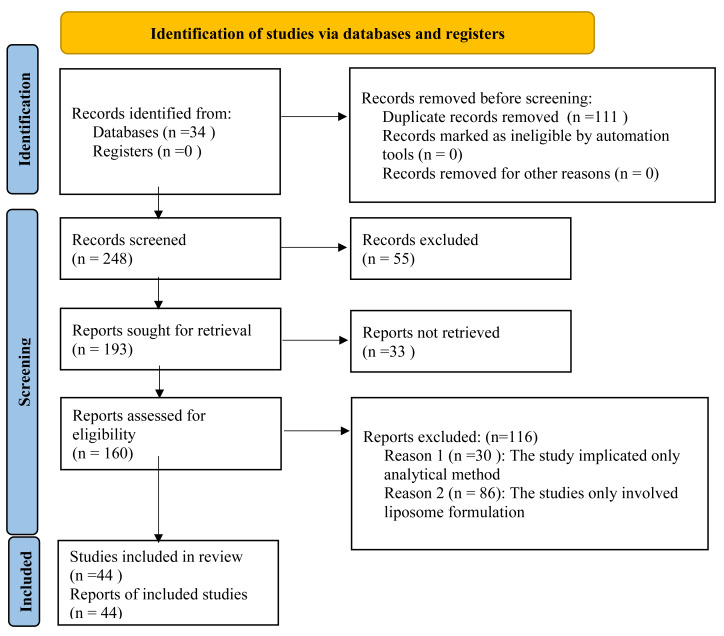
PRISMA flow chart.

The concept of liposomes was initially described by the biophysicist Alec Bangham in the 1960s, when he demonstrated the spontaneous formation of vesicular structures in egg lecithin upon its introduction into an aqueous solution (Leung et al., 2019). The first liposome-encapsulated drug was approved in the United States (USA) in 1995. This medicine, known as Doxil^®^, was developed by Chezy Barenholz and Alberto Gabizon in Israel and the United States to treat patients with ovarian cancer and AIDS-related Kaposi’s sarcoma who had poor outcomes or demonstrated intolerance to the first-line systemic chemotherapy (Barenholz, 2012; Bulbake et al., 2017). Liposomes are artificial microscopic vesicular structures composed of one or more concentric lipid membranes that enclose an internal core with hydrophilic properties. Bilayers are usually composed of phospholipids, where the polar head has direct contact with the internal aqueous core (Inglut et al., 2020; Lamichhane et al., 2018; Nogueira et al., 2015). Figure 1 illustrates the structure of a liposome.

Liposomes are versatile carriers in the pharmaceutical, nutraceutical, and cosmetic industries, facilitating the administration of multiple molecules and enhancing their stability. The instability of some molecules is a challenge and is commonly observed through physical and chemical changes related to temperature, pressure, oxidation, sedimentation, and light degradation (Aditya, Espinosa & Norton, 2017; McClements, 2015; Oliveira et al., 2022). Additionally, conditions during administration, such as gastrointestinal tract (GIT) pH and temperature, can cause degradation, reduced absorption, and reduced bioavailability, particularly for hydrophobic compounds (McClements, 2015; Rideau et al., 2018). Improvements in the stability of some therapeutic agents and membrane similarities between liposomes and cells have led to their consideration as carriers for the delivery of therapeutic compounds, including gene transport (Rideau et al., 2018; Belhadj et al., 2023). Because of their bilayer architecture, liposomes have an ideal environment for the transport of molecules with hydrophobic, hydrophilic, or ionic properties and provide resistance to the previously mentioned physical factors, as well as enhanced internalization, prolonged absorption or circulation time, and specificity in their release sites and routes of administration (Rahman, Uahengo & Likius, 2018; Daraee et al., 2016; Lamichhane et al., 2018; Leung et al., 2019; Nsairat et al., 2022).

Despite all the alternatives and the different advantages of these carriers, they also exhibit difficulties and challenges such as low solubility, low stability, short half-life, oxidation, and hydrolysis of the phospholipids, high production costs, and, in some cases, the induction of immune responses (Daraee et al., 2016; Inglut et al., 2020; Lamichhane et al., 2018; Yu et al., 2021).

For many years, liposomes have been used to deliver medicine to the human body and contribute to human health. Nutraceuticals are a complement or alternative to medicines that have increased in use because of their lower side effects, and many advantages have been demonstrated with liposomes as a delivery system (Naveen & Baskaran, 2018; Ting et al., 2014). In cosmetics, liposomes contain many substances and, due to their physicochemical characteristics, can cross the barriers with facility and deliver the compound locally (Lai et al., 2020; Trauer et al., 2014).

This review was made with the purpose of describing the principles characteristic of liposomes: structure, size, composition, and preparation methods. The advantages of compounds were mentioned in order to improve the stability, permeability, and bioavailability of many substances in the organism. The application of liposomes was summarized in three specific pharmaceutical fields that do not exist in the literature. Generally, revisions in this topic focus on a single domain, but this revision integrates these three fields, which are highly relevant to pharmaceutical areas.

These areas share common technological challenges, such as stability, encapsulation efficiency, controlled release, and scalability, as well as overlapping solutions. Innovations developed in one field are often transferable to other areas. These revisions offer a global overview of the topic, with a focus on applications in the pharmaceutical field.

## Survey Methodology

A bibliographic review was carried out by consulting databases such as ScienceDirect, PubMed, Wiley Online Library, Multidisciplinary Digital Publishing Institute (MDPI), National Center for Biotechnology Information (NCBI), Directory of Open Access Journals (DOAJ), and Nature.

The keywords used were liposomes, liposomes and cosmetics, liposomes and cosmeceuticals, liposomes and nanotechnology, Transferosomes^®^, Ethosomes, liposome-modified vesicles, liposomes and nutraceuticals, liposomes and natural products. The search was conducted in English and Spanish from 2010 to 2025, covering all records available during that period.

**Figure 2:**
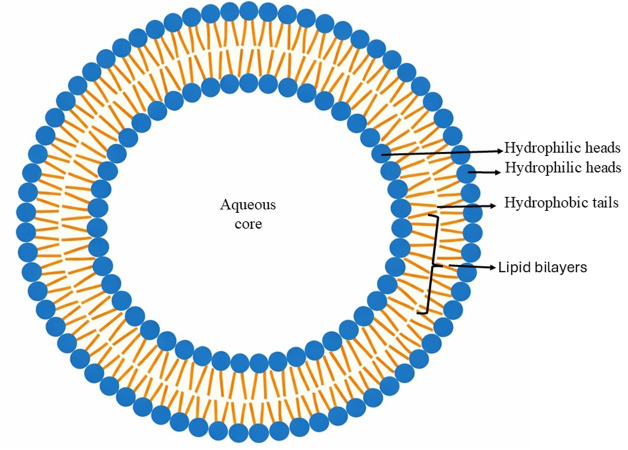
Schematic drawing of the basic structure of a liposome.

The search included original studies, reviews, and book chapters on liposomes, their structure, characteristics, encapsulation methods, therapeutic, nutraceutical, and cosmetic applications, and liposome stability. Articles were selected according to their impact and relevance to the topic. Exclusion criteria included documents without identifiable authors, studies published before 2010, content lacking verifiable information, and works focusing on other systems, such as microemulsions. The study selection process is reported according to the PRISMA 2020 statement (see Fig. 2 and Table S1).

## Overview of Liposomes

Liposomes are spherical vesicles formed by amphiphilic molecules, which contain hydrophilic heads and hydrophobic tails and can be derived from animal or plant sources. They can form by spontaneous self-assembly when phospholipids, the main component, are dispersed in an aqueous medium; this was first reported in 1965 by Alec Bangham and his co-workers (Li et al., 2019; Shah, Ullah & Imran, 2017; Shah et al., 2020). Closed membranes can also form when pure lecithin is agitated in water (Gökçe et al., 2016). These structures are characterized by lipid bilayers with a hydrophilic inner core, and their sizes range from approximately 20 nanometers to several micrometers (Li et al., 2019; Shah, Ullah & Imran, 2017; Shah et al., 2020).

Lipid bilayers can be composed of natural substances that exhibit biocompatibility, biodegradability, inherent low immunogenicity, and low toxicity, because their composition is similar to that of the cell membrane (Lovrić, Knez & Pantić, 2020). For this reason, they have great potential as drug-delivery systems, improving pharmacokinetic and clinical efficacy. Phosphatidylcholine, phosphatidylglycerol, dipalmitoyl phosphatidylcholine, distearoyl phosphatidylcholine, cholesterol, stearyl amine, and diethyl phosphate are among the components commonly used in liposomal formulation (Lovrić, Knez & Pantić, 2020).

Cholesterol plays a vital role in liposome stability. Membrane permeability regulation, fluidity, membrane strength, elasticity and stiffness, transition temperature, drug retention, phospholipid packing, and plasma stability can be enhanced by cholesterol addition. The hydroxyl group of the cholesterol head interacts with the phosphate and carbonyl groups of the phospholipid, causing immobilization of the phospholipid hydrocarbon chains, which increases the rigidity of the lipid bilayer and reduces bilayer permeability. As a consequence, cholesterol-containing liposomes exhibit more stable structural stability and can extend their circulation time for many hours (Aguilar et al., 2012; Pavlovic et al., 2025; Luo et al., 2025).

**Figure 3:**
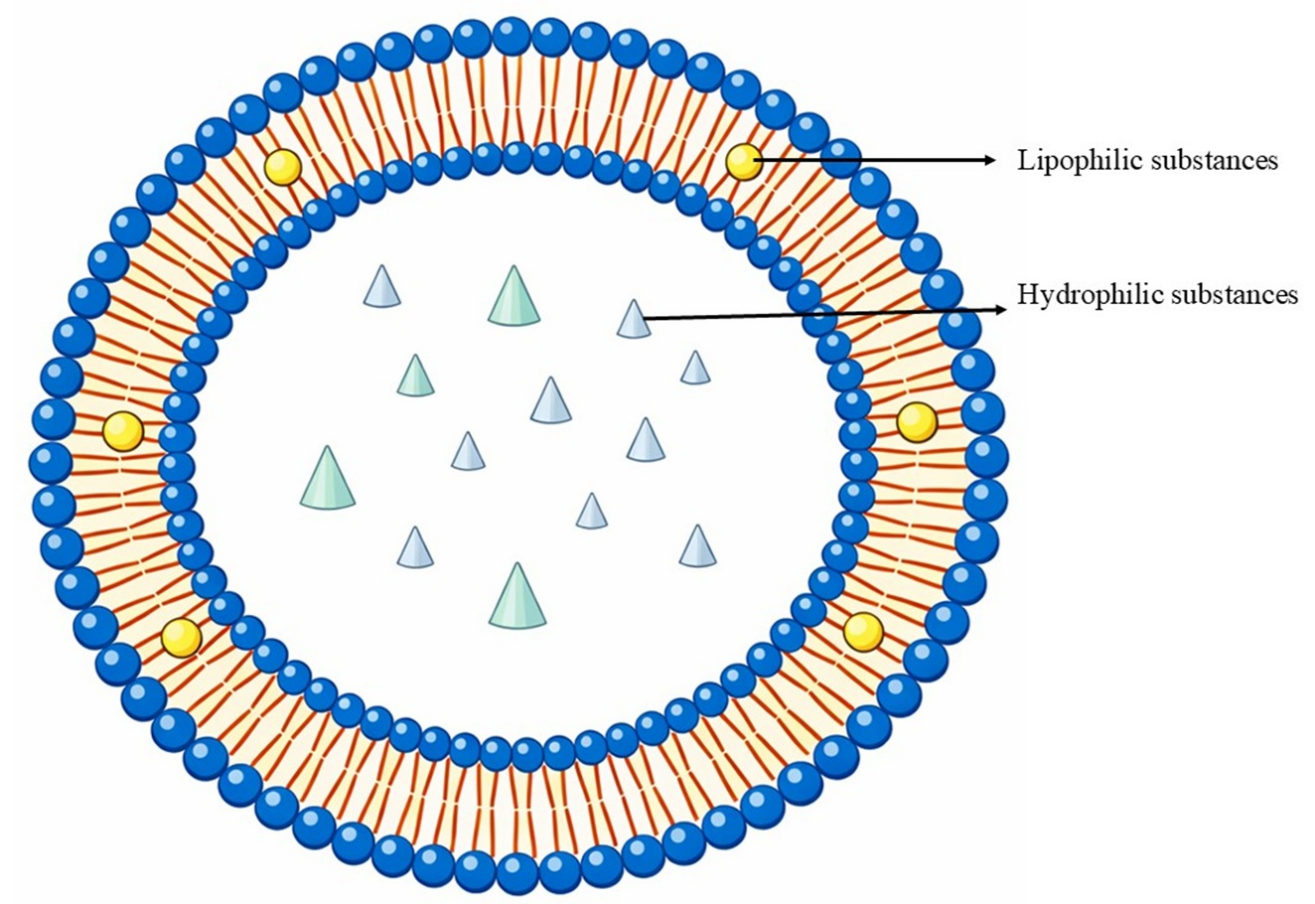
Affinity of different substances according to the bilayer composition of the liposome.

The amphiphilic nature of liposomes allows the encapsulation of water-soluble substances in their aqueous core and hydrophobic substances in the lipid bilayer. Figure 3 shows the arrangement of different substances according to their bilayer affinity. These characteristics confer significant advantages as a delivery system compared with other nanocarrier platforms (Gökçe et al., 2016).

Liposomes are classified based by lamellarity (number of bilayers), size, charge, and functionality. The functionality is conferred by differences in composition and can be subdivided into conventional, long-circulating, cationic, stimulus-sensitive, targeted liposomes, and immune liposomes (Gökçe et al., 2016; Dymek & Sikora, 2022).

## Classification

### Size

The size of liposomes is an important parameter for determining their clinical or therapeutic efficacy. This factor is essential for determining the liposomes’ half-life and the percentage of encapsulated drug. In the medical field, liposomes are typically formulated between 0.025 µm and 2.5 µm (Akbarzadeh et al., 2013; Nogueira et al., 2015).

There are usually two major liposome classifications: unilamellar vesicles and multilamellar vesicles. Unilamellar liposomes are vesicles that present a single phospholipid bilayer; they are subclassified into three categories: small unilamellar vesicles (SUV) (0.02 µm–0.1 µm), large unilamellar vesicles (LUV) (0.1 µm–1 µm), and giant unilamellar vesicles (GUV) (>1 µm).

**Figure 4:**
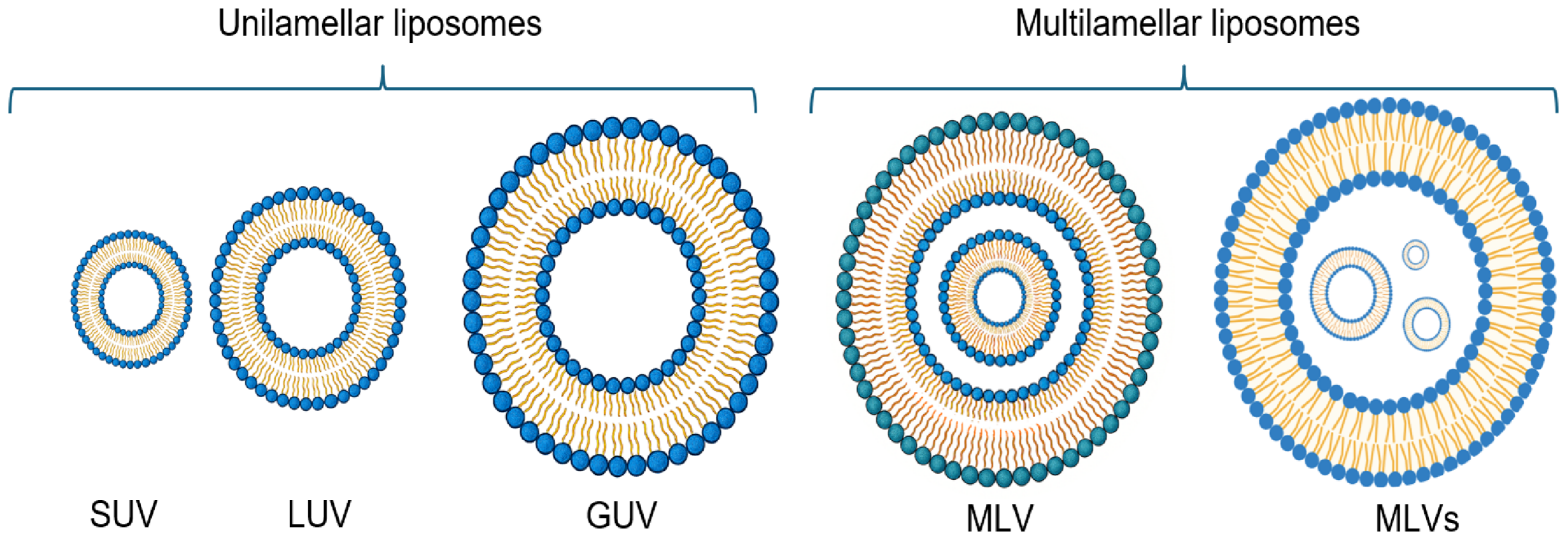
Schematic representation of different classifications of liposomes according to their size.

In addition, there are vesicles with more than one layer or bilayers, such as multilamellar vesicles (MLV) and vesicles containing multiple vesicles called multivesicular vesosomes (MVVs) (Alavi, Karimi & Safaei, 2017; Rideau et al., 2018); Fig. 4 illustrates the differences in size of liposome structures.

The size of liposomes determines important parameters such as circulation half-life and biodistribution, and is associated with lamellarity (number of bilayers), which together influence encapsulation efficiency (Nogueira et al., 2015). The literature reports a wide range of size measurements across liposome classifications, reflecting the distinct functional properties and applications associated with each type.

### Composition

Liposomes have emerged over the years and sparked scientific interest; nowadays, they can contribute to different fields, transporting many substances with high specificity and improving the delivery of many products, protecting them from degradation or premature loss. Table 1 summarizes the main liposome classifications based on composition, their advantages, and associated challenges (Leung et al., 2019; Subramani & Ganapathyswamy, 2020).

**Table 1:** Summaries of different types of liposomes, advantages, and limitations.

**Types of liposomes**	**Description**	**Advantages and limitations**
Conventional Liposome	It was the first liposome used on pharmaceutical preparations. Made of natural phospholipids or lipids (Daraee et al., 2016; Nogueira et al., 2015).	These liposome formulations helped reduce the compound’s toxicity *in vivo* (Daraee et al., 2016; Nogueira et al., 2015). A disadvantage of these liposomes is their rapid elimination from the bloodstream (Daraee et al., 2016; Nogueira et al., 2015).
Long Circulating Liposomes	It’s the result of attaching a hydrophilic polymer to the hydrophilic head group of phospholipids (Saraswat & Maher, 2020; Shah, Ullah & Imran, 2017; Sivadasan et al., 2022).	Reduce liposome aggregation and plasma protein binding. Pegylation reduces the recognition by opsonins and the Mononuclear Phagocyte System (MPS) (Saraswat & Maher, 2020; Shah, Ullah & Imran, 2017; Sivadasan et al., 2022). Liposomes remain in circulation for an extended period (Saraswat & Maher, 2020; Shah, Ullah & Imran, 2017; Sivadasan et al., 2022).
Cationic Liposomes	Composed of DNA, RNA, or antisense oligonucleotides together with cationic lipids (Daraee et al., 2016; Leung et al., 2019; Nogueira et al., 2015).	Used to modify genetically certain target cells because nucleic acids alone can’t cross the cellular lipid bilayer (Daraee et al., 2016; Leung et al., 2019; Nogueira et al., 2015). A problem consists of the generation of toxicity in macrophages and monocytes and can activate the complement system (Inglut et al., 2020; Nogueira et al., 2015).
Stimulus-sensitive Liposomes	Products are released from nanocarriers when a stimulus is applied or when certain conditions are met (Sahandi Zangabad et al., 2018)	The formulation can respond to various stimuli, including pH, heat, light, magnetic particles, ultrasound, and enzymes. Some, such as heat sensitivity, are only adequate under hyperthermic conditions (Abri Aghdam et al., 2019; Chen, Goldys & Deng, 2020; Mura, Nicolas & Couvreur, 2013; Heidarli, Dadashzadeh & Haeri, 2017; Kostevšek et al., 2020; Podaru et al., 2014; Shirmardi Shaghasemi, Virk & Reimhult, 2017).
Inmunoliposomes	Are liposomes with antigens or antibodies inside the vesicles or the bilayer (Daraee et al., 2016; Leung et al., 2019).	Acting as immunological adjuvants on specific target cells, minimizing activity against healthy cells. It’s an excellent alternative for performing immunoassays or diagnostic tests due to the high biodegradability, low toxicity, low antigenicity, and high potential to specific targets (Daraee et al., 2016; Leung et al., 2019; Nogueira et al., 2015).

#### Conventional liposomes

Conventional liposomes were the first liposomes used in pharmaceutical applications. Due to the presence of uncharged or negatively charged lipid compounds, they are subject to endocytosis. Once they are in the cytosol, they have two possible outcomes: to fuse with endosomes or to be delivered to lysosomes for their degradation. They are usually made up of natural phospholipids or lipids such as 1,2-distearyl-sn-glycerol-3-phosphatidylcholine (DSPC), sphingomyelin, egg phosphatidylcholine, and monosialoganglioside; however, they may also contain synthetic phospholipids, like; 1,2-dipalmitoyl-sn-glycero-3-phosphorylethanolamine; 1,2-dipalmitoyl-sn-glycero-3-phosphocholine (DPPC) (Daraee et al., 2016; Nogueira et al., 2015). A significant disadvantage of these liposomes is their rapid clearance from the bloodstream due to uptake by the mononuclear phagocyte system.

#### Long circulating liposomes

These systems, known as “stealth liposomes,” are typically formed by attaching a hydrophilic polymer to the hydrophilic head group of phospholipids. Polyethylene glycol (PEG) is the most commonly used polymer because it prolongs the circulation time of these systems through steric stabilization provided by the polymer coating; this modification reduces liposome aggregation and plasma protein adsorption (Saraswat & Maher, 2020). Moreover, PEGylation also reduces recognition by opsonins and the Mononuclear Phagocyte System (MPS), and allows liposomes to remain in circulation for extended periods (Saraswat & Maher, 2020; Shah, Ullah & Imran, 2017; Sivadasan et al., 2022).

#### Cationic liposomes

Cationic liposomes are composed of DNA, RNA, or antisense oligonucleotides complexed with cationic lipids. These systems are used to genetically modify certain target cells because nucleic acids alone cannot cross the cellular lipid bilayer. Therefore, the relatively simple formulation of these liposomes represents a promising alternative for transporting these compounds; factors such as the presence of polyethylene glycol, surface charge, and number of lipid bilayers in the liposomes influenced their sequestration (Daraee et al., 2016; Leung et al., 2019; Nogueira et al., 2015).

The advantage of having positively charged lipids is that they allow for physical association *via* electrostatic attraction, forming complexes that serve as the structural basis for Lipid Nanoparticles (LNPs). Without this positive charge, the self-assembly process required to effectively encapsulate the genetic material within the delivery vehicle would not occur (Leung et al., 2019; Nogueira et al., 2015).

The positive charge facilitates the formation of intermediate hydrophobic complexes. These complexes, formed between cationic lipids and plasmid DNA, can subsequently be extracted with organic solvents. Functionally, these complexes act as essential intermediates for generating stable particles suitable for gene transfer into cells (Leung et al., 2019; Nogueira et al., 2015).

Cationic lipids such as Trimethyl[2,3-(dioleyloxy) propyl] ammonium chloride (DOTMA) and 1,2-dioleoyl-glycero-3-phosphoethanolamine (DOPE) are often combined with other cationic lipids to facilitate liposome formation. However, one important disadvantage of this type of liposome is that it exhibits toxicity in macrophages and monocytes, alters the release of immune response mediators, and can lead to the generation of free radicals, the overproduction of cytokines, and complement activation (Inglut et al., 2020; Nogueira et al., 2015).

#### Stimulus-sensitive liposomes

Around the 1970s, the development of thermosensitive liposomes for drug release under hyperthermia conditions began; hence, the term “thermosensitive drug ” was coined to refer to this liposome application (Mura, Nicolas & Couvreur, 2013). The nanocarriers that respond to stimuli are designed to trigger the release of the product stored in the bilayer or the aqueous core upon application of one or more stimuli, whether chemical, biochemical, or physical (Heidarli, Dadashzadeh & Haeri, 2017; Lee & Thompson, 2017).

In clinical, therapeutic, and diagnostic applications, stimuli may originate from internal factors such as enzymatic activity, changes in pH, or the presence of reducing agents; they may also be applied externally, such as temperature, light, a magnetic field, or ultrasound (Sahandi Zangabad et al., 2018). There are two main strategies for preparing stimuli-responsive liposomes: one involves the phase transition of the lipid membrane in response to specific stimuli, and the other involves the incorporation of lipids and polymers that modify the structure of the liposomal membrane in response to specific stimuli (Deng, Ling & Li, 2018; Yuba, 2020; Sahandi Zangabad et al., 2018). The types of stimulus-sensitive liposomes are described below.

##### pH-sensitive liposomes.

pH-sensitive liposomes were first used in 1980 to develop a system capable of releasing the encapsulated product under acidic conditions (Abri Aghdam et al., 2019). The concept of these liposomes arose from the observation that some viruses have evolved strategies to enhance infection of cells that contain acid lumens (Paliwal, Paliwal & Vyas, 2015). To develop pH-sensitive liposomes, lipids such as phosphatidylethanolamine or its derivatives are combined with ionizable compounds containing acid-sensitive groups (*e.g.*, carboxylic groups) that stabilize the bilayer under physiological pH (Mura, Nicolas & Couvreur, 2013; Paliwal, Paliwal & Vyas, 2015; Szachniewicz et al., 2024).

Phosphatidylethanolamine (PE) is a phospholipid with a small, poorly hydrated head group that, at neutral pH, forms a cone-shaped lamellar phase. However, in acidic conditions, the molecule is protonated and changes from a conical to a cylindrical shape, facilitating the transition to an inverted hexagonal phase (Paliwal, Paliwal & Vyas, 2015; Rideau et al., 2018). The difference in pH induces morphological changes in the lipid bilayer, increasing liposomal membrane permeability and facilitating the release of the encapsulated products (Lee & Thompson, 2017). 1,2-dioleoyl-glycero-3-phosphoethanolamine (DOPE) is a natural lipid frequently used in the formulation of pH-sensitive liposomes; commonly, it is combined with acid groups such as oleic or succinic acid derivatives, or lipids such as cholesterol hemisuccinate (CHEMS), N-succinyl-DOPE, or dioleoyl phosphatidylcholine (DOPC), in order to induce greater sensitivity to pH (Lee & Thompson, 2017; Mura, Nicolas & Couvreur, 2013; Paliwal, Paliwal & Vyas, 2015).

##### Heat-sensitive liposomes.

Temperature-sensitive liposomes are among the most studied nanosystems for drug delivery, especially in oncology (Mura, Nicolas & Couvreur, 2013). They were first introduced by Yatvin and Weinstein in 1978, when they attempted to inhibit bacterial protein synthesis by releasing neomycin from liposomes at a specific temperature (Aryasomayajula, Salzano & Torchilin, 2017; Ta & Porter, 2013). The release of the content increases or decreases with temperature and retains the encapsulated product at physiological temperature (Mura, Nicolas & Couvreur, 2013). Currently, the study and application of this class of liposomes are focused on the combination with exposure to mild hyperthermia; in this way, temperature-sensitive liposomes are organized in three formulation types (Sahandi Zangabad et al., 2018).

###### Traditional heat-sensitive liposomes.

Traditional heat-sensitive liposomes were further developed in the two decades following their initial introduction. Thermal responsiveness arises from the phase transition of the lipid constituents in the liposomal membrane at a specific temperature, known as the transition temperature (Mura, Nicolas & Couvreur, 2013). The transition phase consists of the change from a gel to a liquid state. In the gel phase, the lipids are ordered and have fully extended hydrocarbon chains, and after heating, the lipid head groups’ mobility gradually increases. Near the transition temperature, the carbon–carbon single bonds between the hydrocarbon chains change from trans to gauche; consequently, holes or pores begin to form due to packing difficulties arising from molecular incompatibilities (Ta & Porter, 2013).

Above the transition temperature, the liposomal bilayer is completely liquid, with lipids freely moving; the membrane becomes fluid and fully permeable (Ta & Porter, 2013). During the transition phase, the encapsulated product leaves the vesicle (Ta & Porter, 2013). Traditional heat-sensitive liposomes are composed of common phospholipids and heat-sensitive components, such as dipalmitoyl phosphatidylcholine (DPPC), a phospholipid with a transition temperature around 41 °C, and the ability to release 20% of the content encapsulated in the liposome (Kim, 2007; Aryasomayajula, Salzano & Torchilin, 2017).

###### Heat-sensitive liposomes with lysolipids.

In 1999, Anyarambhatla and Needham proposed incorporating lysolipids in the membrane of traditional heat-sensitive liposomes to speed up the phase transition and increase the release rate of encapsulated products (Ta & Porter, 2013). Lysolipids are phospholipids with a single hydrocarbon chain and a relatively large head group that provides a positive intrinsic curvature and facilitates micelle formation (Ta & Porter, 2013; Zhang et al., 2011). These lipid bilayer components in a low molar ratio act by two mechanisms: (i) stabilize defects that occur in the phase transition and facilitate the formation of pores in the membrane by forming structures similar to micelles at the membrane edge, and (ii) the lysolipids dissociate from the liposomal bilayer during the transition phase and, as a consequence, generate spaces improving membrane permeability (Banno et al., 2010; Heidarli, Dadashzadeh & Haeri, 2017). The incorporation of lysolipids lowers the phase transition temperature from 43 °C to 39–40 °C and enhances product release over tens of seconds, significantly increasing the percentage of drug release capacity (Sahandi Zangabad et al., 2018; Ta & Porter, 2013).

###### Polymer-modified heat-sensitive liposomes.

The most recent approach to sensitizing liposomes to temperature incorporates natural or synthetic polymers able to change the membrane in response to heat (Ta & Porter, 2013). These polymers exhibit two solubility limits: the Lower Critical Solution Temperature (LCST) and the Upper Critical Solution Temperature (UCST); below and above this temperature, polymers are soluble, and near these temperatures, the structure can fully or partially degrade, or may undergo a structural transition from a coil to a globule (Sahandi Zangabad et al., 2018).

Generally, polymers modify liposome properties at an LCST; at this temperature, structural modification occurs spontaneously and endothermically, driven by the entropy gain from the release of hydrogen bonds with water molecules (Ta et al., 2010). Some of the most studied temperature-sensitive polymers for this application are poly(N-isopropyl acrylamide) (p(NIPAAm)), poly(N-vinyl ethers), elastin-like polypeptides, and poloxamers (Aryasomayajula, Salzano & Torchilin, 2017).

##### Light-sensitive liposomes.

Light-sensitive liposomes were initially reported in 1983. However, it was not until 2000 that the FDA approved the first drug using this technology; the drug Visudyne is a treatment for ocular conditions such as myopia, ocular histoplasmosis, and Age-related Macular Degeneration (AMD) (Chen, Goldys & Deng, 2020). Light-activated technologies have therapeutic uses, such as photodynamic therapy (PDT) for neovascular elimination and angiogenesis in AMD and cancer. Light can control the site and timing of the pharmaceutical ingredient release (Rwei, Wang & Kohane, 2015).

Light acts as an external stimulus, destabilizing the liposomal membrane and releasing its contents. In a successful process, the light source must be adequate to penetrate the tissues (≥ one cm); Miranda & Lovell (2016) note that the near-infrared wavelength range must be selected to ensure penetration of ≥1 cm through the skin and blood. Based on current understanding, five different load release mechanisms can be described:

###### Light-induced oxidation.

The mechanism involves the production of reactive oxygen species (ROS) upon excitation of photosensitizers at specific wavelengths. ROS are compounds containing unpaired electrons and unstable bonds that generate oxidative stress that causes the oxidation of the liposome membrane lipids, pore formation, increased membrane permeability, and enhanced release of the encapsulated product (Bőcskei-Antal et al., 2019; Miranda & Lovell, 2016).

###### Photocrosslinking.

The content release occurs by polymerizing unsaturated bonds in the liposomal bilayer hydrophobic domain *via* irradiation with a specific wavelength of light. The interaction between these molecules causes the bilayer shrinkage and structural conformational changes, leading to the formation of pores, increased membrane permeability, and, consequently, content leakage (Miranda & Lovell, 2016; Yavlovich et al., 2010).

###### Photoisomerization.

Releasing the encapsulated content in a liposome through photoisomerization is achieved by incorporating molecules that undergo conformational changes with light irradiation. In these molecules, isomerization occurs when their spatial orientation changes from trans to cis. This isomerization may increase a molecule’s polarity and decrease its stability, destabilizing the lipid bilayer and releasing liposomal charge. Photoisomerizable molecules most frequently used for liposomal light-controlled release include azobenzene, spirooxazine, stilbene, and spiropyran groups (Leung & Romanowski, 2012; Miranda & Lovell, 2016; Sahandi Zangabad et al., 2018).

###### Photoscission.

Controlled release by photoscission involves photoclearable amphiphilic molecules that are incorporated into the liposomal membrane, specifically into the bilayer hydrophobic region. Light radiation on these molecules with photolabile groups separates the hydrophilic and hydrophobic parts of the amphiphilic phospholipids, leading to loss of surfactant character and facilitating destabilization of the membrane, enabling release of the encapsulated contents (Chen, Goldys & Deng, 2020; Leung & Romanowski, 2012; Miranda & Lovell, 2016).

###### Photothermic release.

The photoinduced thermal release process is based on photon absorption, converting light energy into heat *via* photothermal transducers. The heat generated causes the carried product to be released either by activating heat-sensitive structures that trigger a lipid bilayer phase transition and lead to pore formation, as in traditional heat-sensitive liposomes, or by membrane rupture due to the high tension induced by the high temperature. Nanoparticles of noble metals such as gold, silver, platinum, or palladium are frequently used as photothermal transducers. Gold nanoparticles, especially, have optical and photothermal properties that give them a strong capacity to absorb light and efficiently convert it into heat within a few picoseconds (Alavi, Karimi & Safaei, 2017; An, Zhan & Zhu, 2013; Miranda & Lovell, 2016).

##### Enzyme-sensitive liposomes.

In recent decades, research has been done on developing liposomal systems that respond to the presence of enzymes. It is a special-interest technique in the health field, as it leverages the high expression of enzymes associated with pathological conditions such as inflammation, infection, or cancer (Mura, Nicolas & Couvreur, 2013). In general, the functional design of this class of liposomes is based on the incorporation of polymers, peptides, lipopeptides, or bonds susceptible to the biocatalytic action of a specific enzyme that leads to changes in the liposomal membrane and the consequent release of the encapsulated product (Lee & Thompson, 2017).

Enzyme-sensitive liposomes offer significant advantages over other vesicular systems, particularly when used as drug carriers, since the drug’s release is triggered by an enzyme present in the target tissue. Therefore, these systems do not require external equipment to generate release-activating stimuli; in addition, the risk of side effects is reduced by limiting the area exposed to the drug, and it is even possible to design enzyme-sensitive liposomes that are transformed into bioactive molecules upon enzymatic digestion (Fouladi, Steffen & Mallik, 2017).

Secreted phospholipase A_2_ (sPLA_2_) and matrix metalloproteinases (MMPs) are extracellular enzymes commonly elevated in inflammatory diseases and various types of cancer; they have attracted special interest and have been used to develop enzyme-activated nano-platforms for drug delivery (Aryasomayajula, Salzano & Torchilin, 2017; Pourhassan et al., 2017). sPLA_2_ is an esterase that acts by hydrolysis of the phospholipids incorporated in the liposome, generating fatty acids and lysolipids, breaking the integrity of the lipid bilayer, and releasing the encapsulated product (Fouladi, Steffen & Mallik, 2017). MMPs, on the other hand, are proteolytic enzymes that cleave lipopeptides incorporated into the liposomal bilayer, leading to reduced membrane stability and leakage of the contents (Fouladi, Steffen & Mallik, 2017; Aryasomayajula, Salzano & Torchilin, 2017).

Other extracellular enzymes studied include urokinase-type plasminogen activator, elastase, prostate-specific antigen (PSA), alkaline phosphatase, and transglutaminase; in addition, cathepsin B is investigated for the same purpose, with the difference that it is an intracellular enzyme (Deshpande, Biswas & Torchilin, 2013; Fouladi, Steffen & Mallik, 2017).

##### Magneto-sensitive liposomes.

The combination of magnetic nanoparticles and liposomes was first introduced in 1988 by De Cuyper and Joniau. Magnetosensitive liposomes are systems that contain materials susceptible to magnetic fields, such as metal ions or magnetic nanoparticles, located in the liposome membrane or in the internal core, which act as triggers for controlled drug release. Iron oxide and platinum nanoparticles are commonly used within these nanoparticles and are contained in the liposomal hydrophilic core, lipid bilayer, or attached to the liposome surfaces, depending on their molecular structure (Heidarli, Dadashzadeh & Haeri, 2017; Kostevšek et al., 2020; Podaru et al., 2014; Shirmardi Shaghasemi, Virk & Reimhult, 2017).

Depending on the mechanisms involved, these liposomes can act as fast- or slow-release systems. More specifically, liposomes can participate in hyperthermia mechanisms that depend on heating specific cells, such as cancer cells, with slow release. They can also participate in rapid release mechanisms through exposure to magnetic fields that induce cellular movement. Moreover, these nanoparticles can generate heat under magnetic stimulation, thereby releasing the contained drug (Podaru et al., 2014; Shirmardi Shaghasemi, Virk & Reimhult, 2017).

In both cases, increased temperature induces structural changes in the liposomal membrane, leading to drug release. Liposomes with these characteristics are a promising alternative for cancer treatment due to their ability to achieve site-specific drug release, demonstrated in tumor cells and *in vitro* and *in vivo* tests (Kostevšek et al., 2020; Podaru et al., 2014).

##### Ultrasound-sensitive liposomes.

Ultrasound-sensitive liposomes are of great interest in the medical field due to the ease, effectiveness, and non-invasiveness of extracorporeal ultrasound (Huang & MacDonald, 2004). Ultrasound generates three phenomena: cavitation, hyperthermia, and acoustic transmission. Cavitation governs the response of sonosensitive liposomes, but a response can also be achieved by combining cavitation with hyperthermia (Sahandi Zangabad et al., 2018). There are two main mechanisms for their activation and the subsequent release of their contents:

One of the most studied mechanisms is the incorporation of a gas phase into the liposome, and this action can be carried out through different strategies, such as gas bubble encapsulation, adding a liquid phase that undergoes a physical transition to gas after insonation, or binding bubbles to the vesicle exterior or in the liposome’s proximity (Heidarli, Dadashzadeh & Haeri, 2017). In this case, cavitation can be of two types: internal, low-intensity cavitation, or stable, high-intensity cavitation. It is responsible for generating gas bubbles that burst, causing the formation of pores in the lipid membrane, increasing permeability, or destabilizing and disrupting the liposome (Huang & MacDonald, 2004; Husseini, Pitt & Martins, 2014; Sahandi Zangabad et al., 2018).

The second most studied strategy is heat-sensitive liposomes combined with cavitation and sonication-induced hyperthermia. The release of the encapsulated content from the liposome occurs through the formation of lipid bilayer pores after the liposome undergoes a phase transition, which is activated by the temperature increase when ultrasound is applied (Huang & McPherson, 2014; Husseini, Pitt & Martins, 2014)

Beyond their relevance in minimally invasive biomedical applications, *in vivo* studies suggest that they are potential anticancer agents and contrast agents (Husseini, Pitt & Martins, 2014; Paul et al., 2014).

#### Immunoliposomes

Immunoliposomes are vesicles that contain antigens or antibodies, either within the vesicle or in the lipid bilayer. This type of binding has enhanced the immune response by acting as immunological adjuvants on specific target cells, thus minimizing toxicity against healthy cells during the immune response due to the effect of improved permeability and retention (EPR), which is an effect presented in tumor cells with compounds such as liposomes (Daraee et al., 2016; Leung et al., 2019).

Immunoliposomes are internalized through receptor-mediated endocytosis. The cytotoxic agent is released by endosomal/lysosomal degradation near the target tissue, and then the drug diffuses across the plasma membrane to reach the tissue. This type of liposome offers an alternative for performing immunoassays or diagnostic tests due to its high biodegradability, low toxicity, low antigenicity, and high potential for specific targeting (Daraee et al., 2016; Leung et al., 2019; Nogueira et al., 2015).

## Liposome preparation methods

### Conventional methods

#### Thin-film hydration

is a widely used technique for liposome preparation due to its simplicity; it is also known as the Bangham method. In this technique, the lipids are dissolved in an organic solvent, then the solvent is evaporated, and the lipid film is dispersed in an aqueous medium by agitation at a temperature above the lipids‘ transition temperature. This method allows obtaining liposomes with a small encapsulation capacity for hydrophilic substances but high encapsulation efficiency for lipophilic substances (An & Gui, 2017; Bozzuto & Molinari, 2015; Laouini et al., 2012; Sanarova et al., 2019).

**Reverse-phase evaporation:** this technique involves forming a water-in-oil emulsion between the aqueous and organic phases, then slowly removing the organic solvent under vacuum. The evaporation of the organic solvent yields a liposomal dispersion with a higher capacity to encapsulate hydrophilic material in its core (Nkanga et al., 2019; Laouini et al., 2012; Sanarova et al., 2019; Shah et al., 2020).

**Solvent-injection:** it is a fast and simple technique in which the lipids are dissolved in an organic solvent, usually ethanol or ether, then this lipid solution is injected into an aqueous medium, causing the precipitation of the lipid molecules and the formation of bilayer fragments that are transformed into liposomes (Laouini et al., 2012; Powers & Nosoudi, 2019; Shaker, Gardouh & Ghorab, 2017).

**Detergent dialysis:** this technique for liposome preparation involves the hydration of phospholipids in an aqueous solution containing detergent at its critical micellar concentration, leading to the formation of mixed micelles that ultimately yield liposomes. The detergent is removed through controlled dialysis, absorbent beads, dilution, or column chromatography. This procedure has disadvantages, such as longer preparation time and the formation of liposomes with low encapsulation capacity (An & Gui, 2017; Bozzuto & Molinari, 2015; Nkanga et al., 2019; Laouini et al., 2012; Powers & Nosoudi, 2019).

**Heating method**: This is an attractive liposome preparation technique because it does not require solvents or organic surfactants. The method involves hydration of phospholipids in an aqueous medium and subsequently heating them with a moisturizing agent (glycerin or propylene glycol) to temperatures of 60 °C or 120 °C, depending on whether the lipid mixture contains cholesterol or not (Nkanga et al., 2019; Laouini et al., 2012; Linares-Alba et al., 2016).

### New preparation techniques

The conventional techniques for preparing liposomes are generally simple, fast, and suitable for laboratory-scale operations. However, they present significant disadvantages when scaled up for industrial production. Some disadvantages include those related to the size of the liposomal particles, the stability and toxicity problems associated with organic solvent residues, difficulties in sterilizing liposomal preparations, and a compromise in the technique’s reproducibility (Laouini et al., 2012; Pattni, Chupin & Torchilin, 2015).

With the advances related to the use of liposomes in diverse applications and with the need to produce liposomes with the required quality attributes, some of the conventional production techniques have been improved, as is the case with the solvent injection method, and at the same time, novel techniques have been developed, as the microfluidic channel techniques and the supercritical fluid-based methods (Patil & Jadhav, 2014; Pattni, Chupin & Torchilin, 2015). Some of the manufacturing methodologies currently used are listed below:

-Micro Hydrodynamic Focused (MHF): a technique that allows the self-assembly of liposomes with the possibility of adjusting the size and encapsulation capacity depending on the diameter of the microfluidic channels used.

-Single Hydrodynamic Focusing (SHF)

-Double Hydrodynamic Focusing (DHF)

-Ethanol injection method *via* a micro-engineered nickel membrane.

-Ethanol injection method through a tapered-end glass capillary

-NanoAssemblr and NanoAssemblr Scale-Up Platforms: a system developed by Precision Nanosystems, Canada, to produce liposomes at laboratory and clinical scale using microfluidic technology.

-Supercritical Reverse Phase Evaporation (SRPE): this technique utilizes supercritical CO_2_ (scCO_2_) to dissolve phospholipids.

## Applications of liposomes in different fields

Liposomes, due to their structure and properties, can carry both hydrophilic and lipophilic substances and have multiple applications. Table 2 summarizes some uses of liposomes in pharmaceuticals, nutraceuticals, and cosmetics.

**Table 2:** Applications of liposomes in different fields: drugs, nutraceuticals, cosmetics.

Drug delivery system	Liposomes can be used via many routes of administration, for example, parenteral routes, although the most expensive liposomes have the most drugs approved (Zylberberg & Matosevic, 2016; Wang & Grainger, 2019). In oral administration, liposomes enhance the stability of drugs, protecting the medicine from chemical and enzymatic degradation; improve permeability across the gastrointestinal barrier; and reduce instability in these fluids, which is the most challenging in these routes (Liu et al., 2018). In pulmonary applications, liposomes offer benefits because they have a structure similar to the surfactant and are well-tolerated (Mehta et al., 2020; Rudokas et al., 2016). Liposomes are considered ideal for improving the efficacy of ocular drugs as they helped increase bioavailability (Jacob et al., 2022).
Nutraceuticals	Nutraceuticals have emerged as an alternative and complementary therapy for preventing and treating many diseases (Naveen & Baskaran, 2018; Ting et al., 2014). Awaken interest because they exhibit fewer side effects than synthetic drugs (Naveen & Baskaran, 2018). Curcuminoids have low bioavailability, but when encapsulated in liposomes they can be delivered to exhibit a wide range of biological activities such as anti-inflammatory, anticancer, and antimalarial properties (Aditya et al., 2012; Zheng & McClements, 2020). The relationship between the consumption of olive oil and the low incidence of coronary heart disease is well known (Bonechi et al., 2019; Chin & Pang, 2017; Karković Marković et al., 2019). There are three major components in these oils responsible for these properties: tyrosol, hydroxytyrosol, and oleuropein, but they have low bioavailability and are difficult to reach the target site, liposomes are a delivery system that can solve these problems (Bonechi et al., 2019; Souyoul, Saussy & Lupo, 2018). Flavonoids like quercetin, kaempferol, and luteolin are highly susceptible to degradation and oxidation due to their highly unsaturated structure, and their low solubility and bioavailability further affect their therapeutic action; therefore, they are good candidates for encapsulation in liposomes (Huang et al., 2017; Souyoul, Saussy & Lupo, 2018; Kaushal, Singh & Singh Sangwan, 2022). However, the number of hydroxyl groups in flavonoids can affect lipid bilayer behavior when encapsulated in large quantities (Huang et al., 2017; Souyoul, Saussy & Lupo, 2018).
Cosmetics	Many advantages have been described for the use of nanoparticles in cosmetics; for example, they improve product stability, liposomes retain moisture on their own, do not readily cause condensation or sedimentation, and permeate the stratum corneum (Gökçe et al., 2016; Himeno, Konno & Naito, 2017). Liposomes with a rigid structure pose some difficulty in passing through barriers; therefore, it is necessary to add an edge activator between the phospholipids to impart flexibility or deformability. These kinds of vesicles are ethosomes and transferosomes (Lai et al., 2020; Trauer et al., 2014). Others use hydrophilic or lipophilic additives to provide fluidity and deformability (Jacob et al., 2024).

### Drugs

Liposomes have been studied and used for various medical applications, including anticancer therapy, gene therapy, vaccine delivery, and medical imaging as contrast agents. Liposomes are versatile tools; they can simultaneously deliver both hydrophobic and hydrophilic compounds. Liposomal vesicles have valuable properties that translate into benefits in the pharmaceutical field: they protect the encapsulated product against inactivation and degradation, improve drug bioavailability, and are considered inert and non-toxic (Pattni, Chupin & Torchilin, 2015; Stryjewski, Stefater & Eliott, 2019; Yaroslavov et al., 2015). The use of liposomes as drug carrier systems through different routes of administration will be detailed below:

#### Parenteral

Most approved parenteral liposomal drugs are indicated for intravenous use (Zylberberg & Matosevic, 2016). Compared with conventional parenteral drugs, drug-loaded liposomal systems have some disadvantages, such as high production costs and physical and chemical instability. There are recall reports of liposomal pharmaceutical products from the market due to problems related to long-term storage, instability related to phospholipid oxidation, liposome aggregation or fusion, lack of re-dispersibility, and drug leakage. Currently, lyophilization is the main strategy to improve the shelf life of these pharmaceutical products and reduce stability-related risk, despite being a technique that further increases their manufacturing costs (Wang & Grainger, 2019).

#### Oral

The study of liposomes for oral drug administration dates to the late 1970s. The first oral application of a drug using liposomes was insulin, but this proved neither reproducible nor predictable in terms of efficacy, and it was shelved in the 1980s (He et al., 2019). In recent decades, significant technological advances have facilitated the development of nanodrug carrier systems that have enhanced the bioavailability of orally administered liposomes by improving solubility, a key determinant of bioavailability, and by protecting the therapeutic agent against chemical and enzymatic degradation. However, liposomes for oral use have limitations, including limited gastrointestinal permeability due to the mucosal barrier and instability under gastrointestinal conditions (Liu et al., 2018).

Currently, research is focused on improving the liposome stability and absorption by modifying the liposomal surface; in addition, the functionalization of these systems is being pursued. The methods used to enhance liposomes through surface modification are summarized in four trends: (1) Adsorption of surfactants on the surface to improve stability and sustained oral release, (2) Coating with both natural and synthetic polymers to improve stability, sustained release, and solubility of the drug, and increase adherence to the mucosa, (3) modification of the liposomal surface to obtain multivesicular carriers to improve stability and sustained release, in addition to increasing adherence, and (4) the creation of multivesicular carriers by coating the liposomes with chitosan and β-glycerophosphate to improve stability, sustained release and adherence. All these improvements aim to increase the bioavailability of the encapsulated product (Nguyen et al., 2016).

#### Pulmonary

Liposomal vesicles as pulmonary drug delivery systems have been investigated for the past two to three decades (Mehta et al., 2020). Delivery of inhaled drugs to the lungs is relatively straightforward; however, drug retention in the lungs has always been an obstacle due to high elimination rates, especially for low molecular-weight molecules. Liposomes have received particular attention for pulmonary administration, as they offer non-invasive, sustained drug delivery alternatives. In this way, the drug’s residence time in the lung is increased, thereby improving therapeutic efficacy (Shek, Suntres & Brooks, 1994; Yang et al., 2012).

Liposomes for pulmonary applications offer many benefits, including greater tolerability in the respiratory tract due to their structural similarity to pulmonary surfactants. This aspect is improved by using biodegradable lipids that allow endogenous degradation without toxicity. The size of these systems is so small that they can be encapsulated without difficulty in particles with aerosolization properties, and the reduced size also provides longer adherence to the mucosal surfaces of the pulmonary airways (Mehta et al., 2020; Rudokas et al., 2016).

Many studies have demonstrated the safety of liposomes for pulmonary administration; animal studies indicate that prolonged nebulization of liposomes does not cause any physiological abnormalities in the lungs. It is more important to assess the dose-related toxicity. It should be noted that clinical trials have already been conducted with inhaled liposomes for pulmonary tuberculosis, cystic fibrosis, fungal infections, and other conditions (Mehta et al., 2020; Rudokas et al., 2016).

#### Ocular

Drugs used to treat local ophthalmic diseases are administered *via* three routes: topical ocular, subconjunctival, and intravitreal. Currently, topical administration of drugs is used to treat the anterior part of the eye, while the posterior tissues are treated with intravitreal injections. Liquid formulations of solutions and suspensions are among the most commonly used pharmaceutical dosage forms for topical ocular administration, but they are inefficient due to the biological mechanisms of ocular protection (Diebold et al., 2007; Lajunen et al., 2016).

The need to improve ocular drug efficacy, the search for administration systems with minimal loss during administration, and the need for maximum bioavailability have led to considering liposomes as an ideal tool (Jacob et al., 2022). Liposomes have been studied for the administration of ophthalmic drugs since the 1980s; several routes have been investigated, including topical, subconjunctival, intravitreal, and suprachoroidal. An ideal system for the administration of ophthalmic drugs by a topical route can resist precorneal clearance, providing prolonged contact time and adequate drug absorption. It must have adequate viscosity and pH, cause minimal effects, and be easy to administer (Agarwal et al., 2016). Liposomal vesicles preserve the ease of delivery in liquid form and offer unique characteristics as they are very versatile systems that restrict the activity of the drug at the site of action; they are biodegradable and biocompatible nanocarriers; therefore, they present almost zero toxicity and the variety in activation form, size, and electric charge (Chetoni et al., 2015; Diebold et al., 2007; Mishra et al., 2011). At present, the study of liposomal drugs for ophthalmic application focuses on topical application systems activated by NIR light (Lajunen et al., 2016).

Without a doubt, liposomes, due to their unique properties, have improved drug delivery and are a good system for transporting certain types of medicine. Improve solubility, increase bioavailability, major targeting efficiency, compatibility with many routes of administration are only some of the advantages of this delivery system. This can be confirmed by the increasing number of clinically approved formulations (Bulbake et al., 2017; Lamichhane et al., 2018).

However, despite these advantages, many challenges remain. Long-term physicochemical instability arising from phospholipid oxidation, membrane fusion, and drug leakage compromises shelf life and requires diverse techniques to stabilize the formulation. Large-scale manufacturing remains one of the most difficult processes to resolve; batch-to-batch reproducibility is complicated. Also, it is needed to treat the residual solvent content (Nsairat et al., 2022; Shah et al., 2020).

On the other hand, it is also important to note that regulatory agencies require more rigorous, and therefore more analytical, proof to ensure complete control over critical attributes, which complicates future approval (Zylberberg & Matosevic, 2016; Wang & Grainger, 2019).

### Nutraceuticals

Several common diseases, such as cancer, diabetes mellitus, osteoarthritis, depression, heart disease, and others, are treated with effective treatments that often generate undesired side effects (Aditya et al., 2012; Chan et al., 2004; Dutta, Moses & Anandharamakrishnan, 2018; Huang et al., 2017; Naveen & Baskaran, 2018; Sarris et al., 2018).

Throughout history, numerous cultures, for example, Indian culture, have identified several plants and extracts from natural sources used to treat diverse ailments through Ayurveda medicine. As a result, nutraceuticals, defined as nutritional components that provide physiological or therapeutic benefits beyond basic nutritional needs, have emerged as alternative and complementary therapies for preventing and treating many diseases (Naveen & Baskaran, 2018; Ting et al., 2014).

Because of that, even though synthetic drugs have greater and faster effects, they can present non-desired side effects that could be uncomfortable for patients (Naveen & Baskaran, 2018). Therefore, nutraceuticals awaken particular interest because they generally exhibit fewer side effects than synthetic drugs (Sarris et al., 2018; Ting et al., 2014).

Furthermore, the World Health Organization (WHO) estimates that only 10% of the planet’s plants have been used by humans for medicinal purposes. It is estimated that there are around 2.5–5 million species of plants, providing space for further study and the financing of the different nutraceuticals found in them (Naveen & Baskaran, 2018). There are other sources, such as algae, which could represent a viable option, as they are environmentally friendly and well-adapted to different climatic conditions (Sahni et al., 2019).

#### Difficulties for nutraceuticals

Some of the beneficial properties of nutraceuticals for humans are their antioxidant, anti-inflammatory, antidiabetic, antimalarial, antiulcerogenic, hypoglycemic, hepatoprotective, and potentially antidepressant properties, among many others (Bonechi et al., 2019; Naveen & Baskaran, 2018; Sahni et al., 2019; Sarris et al., 2018; Souyoul, Saussy & Lupo, 2018). However, nutraceuticals encounter various challenges during absorption, which can affect their bioavailability despite their properties.

These factors include poor aqueous solubility, insufficient residence time in the gastrointestinal tract, low stability under physiological conditions (*e.g.*, enzymes, bile salts, pH variability), low intestinal permeability, and susceptibility to rapid metabolic transformation, among others (Souyoul, Saussy & Lupo, 2018). Most of these difficulties are caused by their specific chemical structure and by diverse conditions that affect absorption, such as temperature, acidity, and concentration. All those factors result in deficient or incomplete nutraceutical absorption, which accounts for a large percentage of these (Souyoul, Saussy & Lupo, 2018).

Nutraceuticals administered orally are preferred for preventing or treating chronic diseases due to their ease of administration and greater flexibility in dosing schedules. After administering compounds in this way, the environment and physicochemical conditions of the gastrointestinal tract can greatly influence factors such as bioavailability, stability, and therapeutic efficacy, depending on the chemical structure of the nutraceutical. After absorption through the intestinal wall, first-pass metabolism in intestinal cells and the liver significantly reduces the concentration of bioactive nutraceuticals that reach the systemic circulation (Souyoul, Saussy & Lupo, 2018; Subramanian, 2021).

For this reason, new delivery systems represent a promising alternative for nutraceutical products. Those new alternatives are associated with greater tolerance of the oral route and gastrointestinal conditions, improved solubility, enhanced therapeutic action, and increased resistance to degradation, transport, absorption, and bioavailability (Atrooz et al., 2024). One of the most important systems designed to improve all those mentioned properties is the liposomal system, which will be discussed with various examples below:

#### Liposomes and nutraceuticals

Nutraceuticals originate from a wide range of sources, including amino acids, carotenoids, fatty acids, minerals, and vitamins. Many of these have been encapsulated in liposomes to improve their absorption (Atrooz et al., 2024). Furthermore, it is important to clarify that nutraceuticals may be contained in the internal aqueous compartment of the liposome or in the complex phospholipid bilayer. These types of liposomes are also known as phytosomes (Ting et al., 2014).

##### Curcuminoids.

Curcuminoids are polyphenols from the root of *Curcuma longa,* which have three main components: curcumin, demethoxycurcumin, and bisdemethoxycurcumin. These molecules exhibit low water solubility, low bioavailability, photo- and heat-sensitivity, and a short plasma half-life (Jacob et al., 2024). Liposomes were formulated to overcome these disadvantages and enhance the expression of their biological activities, such as antioxidant, antimicrobial, anti-inflammatory, anticancer, and antimalarial properties (Aditya et al., 2012; Zheng & McClements, 2020).

Aditya et al. (2012) loaded a liposome with curcumin, determined the encapsulation efficiency, and conducted an *in vivo* study in mice infected with malaria to evaluate its effectiveness. In the next paragraph, the procedure is described.

Curcuminoid-loaded liposomes were prepared by modifying the thin film hydration method in a range of different curcuminoid: lipid ratios (1:5, 1:7.5, 1:10). Subsequently, the different mixtures of curcuminoids and lipids were dissolved in a mixture formed by a chloroform:methanol mixture (2:1 v/v); then, solvents were evaporated under vacuum. The lipid film formed was rehydrated with buffered saline (pH 7.4) under continuous agitation for 10 min. The homogeneous solution formed was filtered through 100 nm polycarbonate membranes to obtain liposomes with a uniform size. The encapsulation efficiency was calculated, and liposomal systems were evaluated using *in vivo* tests (Aditya et al., 2012).

The encapsulation percentages obtained ranged from 83.63% to 94.36%, with a maximum at the 1:10 ratio of curcuminoids to lipids, possibly because, as the curcuminoids-lipids ratio increased, the available space for encapsulation decreased. The loaded liposomes release test using a buffered saline solution at pH 7.4 shows biphasic behavior. A rapid phase releases 0%–14% of curcuminoids during the first 3 h, and in the second, slower phase, 15%–37% of the content is released over 48 hours (Aditya et al., 2012).

Those tests demonstrate improvements in curcuminoid protection and stability and show the importance of controlling liposome size, a typical characteristic of extended-release systems used for the administration of nutraceuticals (Aditya et al., 2012; Zheng & McClements, 2020).

The *in vivo* study was conducted in mice infected with malaria to evaluate the antimalarial activity of curcuminoids. Six groups of mice were treated; there was a control group, a group that was treated with curcuminoids orally at 40 mg/kg, and the remaining four groups were treated with liposomes: one group with empty liposomes (liposomes without curcuminoids) and three groups with loaded liposomes at doses of 10, 20, and 40 mg/kg (Aditya et al., 2012).

After 7 days, all the mice developed parasitemia and subsequently died, except those treated with loaded liposomes at 40 mg/kg; in this group, parasitemia was delayed until 11 days, and complete mortality occurred on day 13. These results reveal the possibility of administering curcuminoid-loaded liposomes as a co-treatment with the commercial antimalarial drug artemisinin (Aditya et al., 2012).

Another test was carried out, and after 50 days, a group of mice survived and were cured of malaria; the animals were treated with artemisinin (30 mg/kg) and curcuminoid-loaded liposomes (40 mg/kg). The results indicated that there might be a potentiating or even synergistic effect between artemisinin and curcuminoids when co-administered in liposomes (Aditya et al., 2012; Zheng & McClements, 2020).

##### Tyrosol, hydroxytyrosol, and oleuropein.

Tyrosol, hydroxytyrosol, and oleuropein are the major phenolic compounds from *Olea europaea,* commonly known as the olive tree. Both the tree’s fruits and its oil constitute essential components of the Mediterranean diet. The association between this diet and the low incidence of coronary heart disease and cancer, together with its anti-inflammatory, antioxidant, anti-aggregant, anti-infective, and chondroprotective effects, has generated interest in these nutraceuticals for the treatment of various diseases through a liposome-based delivery system that increases their bioavailability (Bonechi et al., 2019; Chin & Pang, 2017; Karković Marković et al., 2019).

Bonechi et al. investigated the encapsulation efficiency of olive phenolic compounds in liposomes by HPLC-UV analysis.

The liposomes were prepared by the thin-film hydration method with standard solutions of 1,2-dioleoyl-sn-glycero-3-phosphoethanolamine (DOPE) and 1,2-dioleoyl-sn-glycero-3-phosphocholine (DOPC), in a 1:1 molar ratio. Standard solutions of tyrosol, hydroxytyrosol, and oleuropein were prepared and added to total phospholipids to obtain liposomes containing a 1:1 molar ratio. The solutions were dried under nitrogen to remove the solvent, then under vacuum. Finally, the samples were rehydrated with Milli-Q quality water (Bonechi et al., 2019).

They were frozen and thawed nine times. The liposomes were extruded through a 100-nm polycarbonate membrane to control particle size and produce predominantly unilamellar liposomes in the final suspension. The suspension was dialyzed for three hours and stored at 4 °C (Bonechi et al., 2019).

Using HPLC-UV, the encapsulation efficiencies were 4%, 12.4%, and 30.2% for tyrosol, hydroxytyrosol, and oleuropein, respectively. The higher percentage of oleuropein suggests that phospholipids such as DOPE and DOPC may be particularly suitable for the encapsulation of these nutraceuticals. Furthermore, this may indicate that oleuropein is preferentially incorporated into the membrane lipid bilayer, forming a phytosome (Bonechi et al., 2019; Souyoul, Saussy & Lupo, 2018).

In addition, an *in vitro* study was conducted to determine the liposomes’ cytotoxicity and cytocompatibility. The tests were performed in NIH3T3 fibroblasts and human chondrocytes. Three concentrations of nutraceutical liposomes were compared (0.1%, 1%, and 5% v/v). Results indicate that only liposomes at higher concentration (5% v/v) had a significant effect on internalizing antioxidant nutraceuticals into human chondrocytes and allowing their release within the cells or specific receptors, without exhibiting cytotoxic activity (Bonechi et al., 2019).

Despite the need to conduct more advanced clinical studies on this type of liposomes in nutraceuticals, the potential for eventual treatment of osteoarthritis or for reducing the collateral effects of commercial drugs on the market (Bonechi et al., 2019).

##### Quercetin, Kaempferol, and Luteolin.

Flavonoids are a group of secondary metabolites that have gained relevance in the field of nutraceuticals, as they could represent a therapeutic option for inflammatory diseases, cancer, and cardiovascular diseases. Unfortunately, these compounds are susceptible to oxidation and degradation due to their highly unsaturated structure; poor water solubility and low bioavailability can facilitate the loss of their therapeutic action (Huang et al., 2017; Souyoul, Saussy & Lupo, 2018; Kaushal, Singh & Singh Sangwan, 2022).

Due to their antimicrobial, anti-inflammatory, and anticancer activities, flavonoids such as quercetin, kaempferol, and luteolin are considered ideal candidates for liposomal encapsulation. However, flavonoids have the particularity that, depending on the number of hydroxyl groups, they can affect lipid bilayer behavior; when encapsulated in large quantities, they could generate a pro-oxidant effect (Huang et al., 2017; Souyoul, Saussy & Lupo, 2018).

Huang et al. (2017) conducted an experiment to investigate the rational design of liposomal encapsulation technology to efficiently deliver flavonoids in nutraceuticals and functional foods. Egg yolk-based liposomes were formulated containing a mixture of lipids and a non-ionic surfactant such as Tween 80. The liposomes were prepared using the thin-film hydration method (Huang et al., 2017).

This technique consists of dissolving a small amount of flavonoids in two mL of ethanol, with 1.72 g of egg yolk and Tween 80, in a molar ratio of 1:0.72, under magnetic agitation for 30 min. The liposomes were prepared with 1%, 3%, and 5% of flavonoid percentages. Subsequently, the solution was evaporated under vacuum, and the thin layer generated was rehydrated with buffered saline solution at pH 7.4, under constant stirring for 2 h at 55 °C (Huang et al., 2017).

To obtain a homogeneous quantity of liposomes, they were sonicated in a cold bath for 10 min. The final concentrations of egg yolk and Tween 80 were 12.5 g/L and 9 g/L, respectively. The liposomes were stored at 4 °C for analysis of encapsulation efficiency, stability, antioxidant capacity, and lipid peroxidation assays (Huang et al., 2017).

Several days after formulation, samples with different concentrations were analyzed, and it was observed that the flavonoids’ storage capacity had an inverse-proportional relationship with the percentage of flavonoids contained. Liposomes containing 5% flavonoids showed low resistance because high concentrations increased the proportion of flavonoids embedded in the membrane, increased liposome size, and altered their stability and behavior. On the other hand, liposomes loaded with 1% and 3% of flavonoids showed high storage capacity. The flavonoid with the greatest storage stability was quercetin, followed by luteolin, and lastly kaempferol (Huang et al., 2017).

The liposomes’ exposure to pH values ranging from 2.5 to 8.5 and temperatures between 20 °C and 80 °C affects their storage capacity and size. Liposomes containing 1% and 3% flavonoids showed minimal variation, but liposomes loaded with 5% flavonoids had a higher variation in the same conditions. It is explained by variation in lipid membrane orientation, for example, with the protonation or deprotonation of phenolic groups at acidic or basic pH, respectively (Huang et al., 2017).

The encapsulation efficiencies were approximately 90% for quercetin and 80% for kaempferol and luteolin. Moreover, the encapsulation efficiency decreased as the flavonoid content increased; thus, liposomes with higher encapsulation capacity contained 1% flavonoids.

The percentages of antioxidant capacity obtained in a DPPH test for quercetin, kaempferol, and luteolin were 73.97%, 60.07%, and 53.73%, respectively. Furthermore, equal behavior was observed in the lipid peroxidation test, with a smaller change in quercetin liposomes and a greater change in luteolin liposomes (Huang et al., 2017).

The orientation of the flavonoid molecules helps explain the observed results; these orientations affect the liposomes’ lipid bilayer characteristics. Quercetin increased the rigidity of the hydrophobic region, and the inward orientation of the luteolin membrane facilitated steric hindrance to radical diffusion and reduced their reaction (Huang et al., 2017).

Finally, it was observed that the proportion of flavonoids influences liposomal behavior, with lower flavonoid concentrations improving encapsulation efficiency, stability, and antioxidant capacity (Huang et al., 2017).

As with liposomes in medicines, liposomes in nutraceuticals offer multiple benefits, including enhanced solubility, stability, and bioavailability of nutraceutical compounds. However, in gastrointestinal conditions, major challenges remain to be solved. Most conventional formulations are susceptible to pH fluctuations, bile salts, and enzymatic hydrolysis during the gastrointestinal transit. These produce rapid structural disintegration and reduce bioavailability (Jahangir et al., 2022; Subramani & Ganapathyswamy, 2020).

Multiple works have been conducted to modify liposome structure and enhance bioavailability; however, there is a need to determine the stability of nutraceuticals in these new liposomes. Industrially, there is a need for large-scale continuous production capability, batch-to-batch irreproducibility, a lack of effective sterilization methods, liposomes generally degrade under heat treatment, and, due to their size, they are not good candidates for procedures like filtration. Elevated costs and the need to ensure human health safety for every compound utilized in the manufacture, because it frequently contains traces of liquid solvents that are toxicological concerns and need to be addressed to comply with regulatory standards (Shukla et al., 2017; Jahangir et al., 2022; Latrobdiba, Fulyani & Anjani, 2023).

### Cosmetics

Cosmetics are regulated by the US Food and Drug Administration (FDA) under the Federal Food, Drug, and Cosmetic Act (FD&C Act). This law defines cosmetics as “articles intended to be rubbed, poured, sprinkled, or sprayed on, introduced into, or otherwise applied to the human body for cleansing, beautifying, promoting attractiveness, or altering the appearance” (US Food and Drug Administration, 2024). Skin moisturizers, perfumes, lipsticks, fingernail polishes, eye and facial makeup, hair colors, and deodorants are among the products included as cosmetics.

Around 1984, the term “cosmeceuticals” appeared to categorize products that fall between the definition of cosmetics (intended to clean or beautify) and pharmaceutical products defined by the FDA as “articles intended for use in the diagnosis, cure, mitigation, treatment, or prevention of disease or that affect the structure or any function of the body”. For this reason, Dr. Albert Kligman described this category as “a topical preparation that is sold as a cosmetic but has characteristics that suggest pharmaceutical action”. Although it is a term that has not been formally recognized by either the FDA or the European Union, it has been widely used in dermatological literature for several years (Pandey, Jatana & Sonthalia, 2025). The FDA only recognizes a product as a drug, a cosmetic, or a combination of both (Pandey, Jatana & Sonthalia, 2025).

Pandey, Jatana & Sonthalia (2025) point out the different indications of cosmeceuticals; among them are anti-aging, photo melanosis and photobronzing treatment, pigment-related disorders, wrinkle reduction, anti-inflammatory effects, fat reduction, promoting hair growth and preventing hair loss, maintenance of skin tone and skin clarity, reduction of scars, acne, rosacea, melasma, moisturizing agents, sunscreens and antioxidants.

These products may improve or conceal specific skin characteristics while also ensuring pharmacological activity, whether local or systemic; therefore, achieving adequate skin absorption is essential to achieve the desired effects (Pandey, Jatana & Sonthalia, 2025).

To achieve optimal absorption, it is necessary to consider the skin as a biological barrier that protects against harmful external agents, prevents the entry of microorganisms, and prevents water loss, among other functions. The skin is composed of three layers (epidermis, dermis, and hypodermis). The superficial layer is the epidermis, composed of keratinocytes, and it is divided into strata (corneum, lucidum, granulosum, spinosum, and basal). The stratum corneum is the outermost protective layer, composed of approximately 10–15 layers of flattened, hexagonal, dead, and cornified cells, each measuring 10–15 µm; these cells, called corneocytes, are surrounded by a lipid-enriched intercellular matrix. They are cells with high levels of keratin and lipids but a low water content of around 15–20%. Substances such as free fatty acids, cholesterol, and long-chain ceramides are found in the lipid matrix. For this reason, effective penetration of active substances through the skin membrane is challenging (Lai et al., 2020).

The transdermal release and the permeability of molecules through the skin occur *via* two main routes. The transepidermal route may follow an intercellular pathway, diffusing between corneocytes *via* the lipid matrix, or a transcellular pathway, crossing corneocytes directly; it allows greater diffusion of lipophilic substances. Alternatively, the skin appendages, such as sebaceous glands, sweat glands, or hair follicles, allow the penetration of substances that cannot cross the stratum corneum due to their hydrophilic nature, thereby facilitating permeation, mainly because of the presence of ions or long polar molecules (Dominguez, Mackert & Dobke, 2017; Lai et al., 2020).

Nanotechnology has become a scientific field with multiple applications, including the food industry, electronics, cosmetics, drugs, and personal care products. It has been demonstrated that nanoparticles exhibit a greater ability to permeate the physical and chemical skin barrier *via* skin appendages. Therefore, some strategies use nanotechnology to improve the release and permeation of active compounds, for example, by reducing particle size without losing their biological activity using lipidic surfaces, such as liposomes (Dominguez, Mackert & Dobke, 2017).

The use of nanoparticles in cosmetic formulations offers multiple advantages, including improved product stability. Particle size is related to stability because the larger the particle size, the more significant the effect of gravitational force will be, and for this reason, precipitation is lower, and shelf-life is longer with nanoparticles than microparticles (Gökçe et al., 2016). Liposomes have become very attractive in cosmetics because they retain moisture on their own, thanks to their main components; for example, one phosphatidylcholine molecule can be hydrated by 10 water molecules. Also, it is preferable to use small, multilamellar liposomes in the cosmetic industry due to their structural stability, as they do not readily cause aggregation or sedimentation and permeate the stratum corneum (Himeno, Konno & Naito, 2017).

Despite the advantages described and the deeper penetration into skin layers that nanocarrier-based release systems enable, conventional, rigid liposomes cannot permeate deeply enough and tend to accumulate in the stratum corneum as a reservoir. Therefore, new liposomes with flexible or deformable properties have been developed using additives that enhance their ability to penetrate deeper layers (Lai et al., 2020; Trauer et al., 2014). Liposomes can be classified into various types depending on the modifications in their structure, which will be discussed below:

#### Transferosomes^®^

Cevc and Blume first introduced Transferosomes^®^ in the early 1990s as a new type of ultra-deformable vesicle. Since then, three generations of Transferosomes^®^ have been developed. The elastic property is introduced by inserting an edge activator between the phospholipids, thereby destabilizing the bilayer. Two mechanisms have been described to explain how permeability is increased; the first indicates that Transferosomes^®^ can pass through smaller pores than its size by adding an edge activator to the vesicle, allowing it to modify its shape. The other mechanism is based on the trans-epidermal osmotic gradient formed by the difference in water content between the skin surface and the interior, indicating that the permeation of Transferosomes^®^ is driven by this force (Caddeo et al., 2018; Lai et al., 2020; Bahia et al., 2009).

Among the most widely recognized edge activators are sodium deoxycholate, Span 60, Span 65, Span 80, Tween 20, Tween 60, Tween 80, and dipotassium glycyrrhizinate; ethanol is sometimes added to these edge activators in small amounts, about 10% or less (Lai et al., 2020). According to the three generations developed, the first generation consists of phospholipids and surfactants, and the second generation focuses on solubilization assays to select the optimal pair of substances between the edge activator and the phospholipids. The newest, third generation corresponds to adaptable vesicles due to amphipathic molecules other than phospholipids (Cevc, 2012; Lai et al., 2020). It should be noted that the advantages of Transferosomes^®^ for skin penetration also depend on their application, which must be non-occlusive.

Comparative studies have been conducted between rigid and flexible liposome formulations. El Maghraby, Arafa & Essa (2018) reported that, to verify the increase in permeability conferred by liposome structures, thermal tests were performed. Furthermore, some of the active substances formulated in Transferosomes^®^ in the cosmetic field include tocopherol, a phenolic compound with antioxidant and photoprotective activity that reduces oxidative damage caused by UV rays. Another substance is resveratrol, a non-flavonoid polyphenol with healing, anti-inflammatory, antimicrobial, and antiviral properties, with applications in diseases such as psoriasis and skin infections, as well as in the prevention of skin cancer. Some formulations include resveratrol and 5-fluorouracil to enhance the anticancer effects of these two compounds, acting synergistically (Caddeo et al., 2018; El Maghraby, Arafa & Essa, 2018; Scognamiglio et al., 2013).

#### Ethosomes

These vesicles are called ethosomes because they contain ethanol at high concentrations (20–50%), along with phospholipids and water. Ethanol is found in large quantities around the hydrophilic heads, providing greater fluidity and flexibility that improves vesicle permeability through the skin membranes. Other studies indicate that ethanol alters the bilayer structure of the stratum corneum. They are characterized by their capacity to retain the active ingredients they carry for longer periods and are smaller and more stable at room temperature than Transferosomes^®^. Ethosomes are stable for up to one year under storage conditions and can be applied occlusively or non-occlusively, unlike previous formulations. The ethosomes formulation may contain other excipients, such as cholesterol, which provides stability to the bilayer, and, for the gel formulation, can use Carbopol, Pluronic F-127, and Poloxamer 407. Azone, lauryl alcohol, and menthol are enhancers of epidermal penetration that have been added in specific formulations. There are two conventional methods for preparing ethosomes: hot and cold; however, the process can be carried out either by the classical mechanical dispersion method or by encapsulation using a transmembrane pH gradient. Among the disadvantages of ethosomes, they are unstable due to oxidative degradation; in production, yields are low (Costa & Santos, 2017; Niu et al., 2019; Zhou et al., 2018).

In 2010, a new type of ethosome known as binary ethosomes was developed by partially replacing ethanol with other alcohols such as propylene glycol or isopropyl alcohol. Two years later, Transethosomes were developed and are a combination of ethosomes and Transferosomes^®^, incorporating the main ethosomes’ compounds and an edge activator agent (Lai et al., 2020). Ethosomes have been studied as carriers of substances such as vitamin E (tocopherol) to improve photoprotection, and they have shown good intracellular and dermal accumulation. Another substance used in ethosome formulations is psoralen, a natural furocoumarin, used for the treatment of vitiligo and psoriasis (Costa & Santos, 2017; Touitou & Godin, 2008; Zhang et al., 2014).

#### Vesicles modified with hydrophilic additives

This type of vesicle uses hydrophilic compounds, such as polyethylene glycol, and penetration enhancers (PEs), such as Transcutol (diethylene glycol monoethyl ether), which improve bilayer properties and affect the intercellular lipid matrix. Other agents, such as Labrasol and glycerol, are used to formulate glycerosomes by replacing more than 10% of the aqueous phase of the liposomal medium, thereby providing greater fluidity and deformability, depending on the glycerol concentration added (Lai et al., 2020).

#### Vesicles modified with lipophilic additives

For cosmetic application, Lai et al. (2020) indicate that Yamazaki et al. (2018) elaborated liposomes containing phytosterol derivatives (cholesterol analog triterpenes) (Mohammadi et al., 2020). Other scientists mention certain terpenes such as limonene, cineol, fenchone, and citral. All these changes are made to enhance skin permeability (Lai et al., 2020).

Liposomes offer multiple benefits for the delivery of cosmetics across the skin. It has significantly enhanced the penetration, stability, and controlled release of active ingredients across the skin barrier, enabling functional and aesthetic benefits. But despite these advantages, several obstacles remain that limit their industrial-scale manufacture.

Production costs are higher than those of conventional cosmetics because of the need for high-purity lipids, sterile manufacturing conditions, and advanced encapsulation or homogenization equipment (Costa & Santos, 2017; Shaat et al., 2025). Moreover, liposomes in cosmetics often require surfactants and pH adjustments, which can destabilize the vesicle membrane and reduce efficacy (Himeno, Konno & Naito, 2017; Oliveira et al., 2022).

From a regulatory perspective, liposomes in cosmetics have led to increased scrutiny of the nanomaterials used. In these fields, manufacturers must comply with safety assessments that include particle size characterization, studies on skin penetration, and toxicological tests; these can add complexity and increase costs (Oliveira et al., 2022; US Food and Drug Administration, 2024).

Future developments will likely involve greener lipid sources, solvent-free manufacturing techniques, improved vesicle stabilization mechanisms, and closer collaboration between academia and industry to translate advanced liposomal carriers into safe, effective, and economically viable cosmetic products.

## Conclusions

Liposomes are artificial, microscopic vesicular structures composed of one or more concentric lipid membranes enclosing an internal aqueous phase. Liposomes have the advantage that they can contain both hydrophilic and lipophilic substances. They have been widely used as vehicles for molecules that pose challenging problems due to their chemical structure and physicochemical characteristics, which, under diverse critical conditions, such as humidity, temperature, or light exposure, which can lead to degradation of these vulnerable substances.

The incorporation of sterols, such as cholesterol, and even phytosterols, enhances membrane stability and confers numerous advantages, including increased fluidity, elasticity, and drug retention. Liposomes have evolved from simple carriers to more sophisticated systems, including those able to respond to physiological or environmental conditions. Although liposomes are promising therapeutic delivery systems, many limitations and specific problems for each route of administration still need to be addressed.

Nutraceuticals typically exhibit low stability and high degradation due to various chemical and environmental factors, which is why liposomes have provided a solution to these problems and substantially improved their protection, stability, bioavailability, and absorption, encompassing numerous formulation variants adapted to the diversity of compounds within this group.

The current focus is on developing scalable, more sustainable formulations and optimizing oral delivery. All these efforts must progress within a regulatory framework, with the purpose of obtaining a formulation that meets the quality requirements to ensure human health while maintaining the benefits.

Finally, in the cosmetic sector, numerous formulations incorporate active ingredients with pharmacological properties, making enhanced dermal absorption an important goal. Liposomes provide a valuable platform for transporting and protecting sensitive compounds, and their use has contributed to improving the penetration of active ingredients into deeper skin layers. However, for these formulations, adjusting the pH and the need for surfactant, can destabilize the vesicle membrane and reduce efficacy; moreover, regulatory authorities are concerned about the use of nanomaterials, which pose important challenges.

Looking ahead, future research on liposomal delivery systems should prioritize improving long-term stability, optimizing large-scale manufacturing, and reducing production costs, while enhancing performance across oral, dermal, and systemic applications. Collectively, these advancements will support the responsible and efficient translation of liposomal technologies in the pharmaceutical, nutraceutical, and cosmetic sectors, consolidating their role as versatile and reliable delivery systems.

##  Supplemental Information

10.7717/peerj.21198/supp-1 Supplemental Information 1 PRISMA checklist

## Additional Information and Declarations

### Data Availability

The following information was supplied regarding data availability: This is a Literature Review.
